# TPP (totally preperitoneal) making single incision laparoscopic inguinal hernia repair more feasible: a comparison with single incision laparoscopic totally extraperitoneal hernioplasty (SIL-TEP)

**DOI:** 10.1186/s12893-024-02372-9

**Published:** 2024-03-05

**Authors:** Qing Huang, Xiaojun Wang, Xionghua Xiang, Changlei Qi, Ting Fei, Encheng Zhou

**Affiliations:** 1 Emergency Department of The First Affiliated Hospital of Ningbo University, 59 Liuting Road, Ningbo, Zhejiang 315010 China; 2 Department of Gastrointestinal Surgery, The First Affiliated Hospital of Ningbo University, 59 Liuting Road, Ningbo, Zhejiang 315010 China

**Keywords:** Totally preperitoneal hernioplasty (TPP), Totally extraperitoneal (TEP), Single incision laparoscopic totally preperitoneal hernioplasty (SIL-TPP), Single incision laparoscopic totally extraperitoneal hernioplasty (SIL-TEP), Inguinal hernia

## Abstract

**Background:**

Totally preperitoneal hernioplasty (TPP) is a concept which was introduced for distinguishing with totally extraperitoneal (TEP). There is few evidence reflecting the single incision laparoscopic totally preperitoneal (SIL-TPP) characteristic. The aim of study is to demonstrate the feasibility of single incision laparoscopic totally preperitoneal hernioplasty (SIL-TPP) and compare the outcomes with the single incision laparoscopic totally extraperitoneal hernioplasty (SIL-TEP) technique.

**Methods:**

During August 2018 and July 2022, 200 inguinal hernia patients received SIL-TPP and 56 patients received SIL-TEP in the First hospital of Ningbo university. The demographics, clinical characteristics, intraoperative and postoperative parameters were retrospectively analysed.

**Results:**

SIL-TPP and SIL-TEP hernia repair were successfully conducted in all patients. There was no conversation happened in two group. Patients’ demographics were comparable when compared between the two groups adding the comparison initial 52 cases analysis (*P* > 0.05). The mean unilateral hernia operative time was significant shorter in the SIL-TPP group than SIL-TEP group (unilateral: 81.38 ± 25.32 vs. 95.96 ± 28.54, P: 0.001). Further study of unilateral hernia operative time revealed the mean indirect hernia operative time was significant shorter in the SIL-TPP group than SIL-TEP group (indirect: 81.38 ± 25.33 vs. 95.87 ± 28.54, P: 0.001). The unilateral hernia operation time trend of initial 52 cases of two group analysis revealed the operation time of SIL-TPP reduced faster than SIL-TEP along with treating number increasing (Figs. 2 and 3). The comparison of initial equal quantity unilateral hernia patient mean operative time revealed the SIL-TPP group was significant shorter than SIL-TEP group (85.77 ± 22.76 vs. 95.87 ± 28.54, P: 0.049). The rate of peritoneum tearing of SIL-TPP group was significant high than SIL-TEP (*P* = 0.005).

**Conclusion:**

SIL-TPP hernia repair is a superior procedure and possess its own distinguished advantages. We recommend it rather than SIL-TEP for treating inguinal hernia, especially for indirect hernia. However, large-scale randomized controlled trials comparing SIL-TPP and SIL-TEP are needed to confirm these results.

## Introduction

Several hernia repair surgeries have been generated by surgeons over the last century. Totally extraperitoneal (TEP) and transperitoneal hernioplasty (TAPP) are considered standard procedure for laparoscopic inguinal hernia repair [1]. Since the first single incision laparoscopy totally extraperitoneal hernioplasty (SIL-TEP) reported by Filipovic-Cugura et al. in 2009 [2], the surgical technique has become increasingly popular for its benefits, such as cosmesis, pain reduction and rapid recovery. Furthermore, many evidence have proved the feasibility and safety of the procedure [3–5]. The most significant difference is TEP procedure prevents entering the peritoneal cavity, but it involves limited working space, visual field and instruments collision. Consequently, expanding the working space and establishing clear vision are significant for SIL-TEP.

Consideration abdominal wall is made up by nine layers, we previous tried and succeed to conducted the TEP procedure all in preperitoneal space (PPS). In addition, we introduced totally preperitoneal hernioplasty (TPP) concept for distinguishing TEP. For our operation was conducted through single incision parallel and distinguishing to the SIL-TEP, the procedure was named by SIL-TPP. Moreover, our results suggested SIL-TPP is a safe and feasible procedure with acceptable short-term outcomes [6]. Up to now, there was no randomized clinical trial comparing SIL-TPP and SIL-TEP inguinal hernia repair. In addition, there is few evidence reflecting the SIL-TPP characteristic or comparing to other hernia repairing procedure.

The aim of this study was to evaluate the safety and feasibility of SIL using both TPP and TEP approaches and compare the procedure characteristics of the two groups.

## Methods

### Patients

During August 2018 and July 2022, 200 inguinal hernia patients received SIL-TPP and 56 patients received SIL-TEP in The First Affiliated Hospital of Ningbo University. All surgeries were performed after obtaining informed consent from the patients. This study was approved by our hospital Institutional Review Board. All patients received physical examination for diagnosing in outpatient clinic, and received an ultrasonogram or abdominal Computed Tomography (CT) if necessary. All inguinal hernia patients enrolled into our institute were considered for SIL-TEP hernia repair in initial stage of study and considered for SIL-TPP hernia repair in intermediate and final stages. Exclusion criteria were as follows: (1) patients age < 20 years, (2) patients combined with cardiopulmonary function or others cannot bear general anesthesia. All the operations were conducted by a single surgical team.

The demographics, clinical characteristics, intraoperative findings and postoperative course of patients were prospectively recorded during study. Patients did not receive antibiotic prophylaxis routinely. If the estimated operation time longer than 2 h, the patient received indwelling urinary catheter before operation. The surgical procedure was conducted with conventional surgical instruments including conventional 30-degree laparoscope (STORZ, Germany). The single-ports used in our study were parallel to our previous description. The main anatomic landmarks were identified including the pubic bone, inferior epigastric vessels, anterior superior spine, Cooper’s ligaments. Patients conventionally received postoperative intravenous COX2 analgesic. Here, patients who previously have received lower abdominal surgery in our operation area, recurrent inguinal hernia and incarcerated hernia cases were regarded as time-consuming cases for the reason that these cases would consume more operation time. Operative time was counted from skin incision to fascial closure. Hernia patients’ operation time were recorded according to the date of surgery. The operation time were list according to the date of surgery thus assessing the operation time trend.

### Surgical technique

Patients were in supine position with arms adducted if necessary and received endotracheal general anesthesia. The surgeon and camera operator stood on the offside of the inguinal hernia. The monitor was placed on the side of the hernia and at the foot patients. In procedure, patient was placed in a Trendelenburg position and the hernia opposite side was tilted down. In SIL-TPP group, the procedure including setting single incision were conducted as our previous study [6]. Setting a 2 cm single preperitoneal incision and insert a single-port device. In the SIL-TEP procedure, a 2.0 cm single skin incision around hernia side umbilicus for unilateral hernia or midline skin incision around umbilicus for bilateral hernia was made. Exposure and incise the anterior sheet of the rectus abdominis 2 cm in length. Expand and insert a single-port device into the space. The conventional instruments were used for operations. The CO2 insufflation pressure was set at 11 mmHg thus creating pneumopreperitoneum. The division procedure and scope of two group was parallel. The sac was reduced in all cases. However, for sac reduction difficult cases, the sac was cut off at the internal inguinal ring. The hernia sacs were routinely separated freed from spermatic cord more than 5 cm. Round ligament of uterus was routinely reserved in female patients. Small peritoneal tears were neglected. Larger peritoneal tears were closed with Hem-o-lok clips or through suturing. 10 cm (craniocaudal) × 15 cm (latero-lateral) size of mesh covering myopectineal orifice was placed into PPS without fixation. The pneumoperitoneum was deflated carefully to avoid displacing the mesh. Close the incision according to the layer.

### Statistical analysis

The analysis included descriptive statistical methods. Patient characteristics between the two groups were compared with the Chi-square or Fisher’s exact test (for expected frequencies < 5) for categorical variables, and the Student’s t or Median test (for the sample size smaller than 30) for continuous outcomes. P value < 0.05 was considered as statistically significant. Linear analysis was used for the trend analysis of operation time along with treating number increasing. P value < 0.05 was considered as statistically significant.

## Results

### All patient analysis

SIL-TPP and SIL-TEP hernia repair were successfully conducted in all patients. There was no conversation happened in two group. Comparative demographics of all patients between SIL-TPP group and SIL-TEP group are described in Table 1. The SIL-TPP and SIL-TEP groups were comparable regarding to age, Body Mass Index (BMI), median American Society of Anesthesiologists (ASA), main type of hernias. The cases of two site of hernias were significant different when compared between the two group (*P* = 0.02). Comparative perioperative data was shown in Table 2. The mean unilateral hernia operative time was significant shorter in the SIL-TPP group than SIL-TEP group (unilateral: 81.38 ± 25.32 vs. 95.96 ± 28.54, P: 0.001). The mean bilateral hernia operative time was also shorter in the SIL-TPP group than SIL-TEP group though no statistical significance (bilateral:114.10 ± 34.25 vs. 122.75 ± 34.74, *P* = 0.631). The postoperative outcomes were shown in Table 3. The postoperative complication rate was comparable (*P* = 0.399). The postoperative hospital of SIL-TPP was significant shorter than SIL-TEP (2.45 ± 1.59 vs. 3.20 ± 2.46). Other parameters such as seroma, hematoma, wound infection, incisions liquid exudation, incision hematoma, mesh infection, upper respiratory infection, urinary retention, urinary tract infection, hydrocele of testes, 24 h visual analogue scale score were similar between groups (*P* > 0.05). All of them were treated conservatively. There was one female suffered recurrence in SIL-TPP group who reserved the round ligament of uterus. The recurrence time was about 1 year later after operation and she received lichtenstein tension-free hernioplasty. No major complications occurred in neither of groups.

**Table 1:** The characteristics of patients and hernias

Characteristics	SIL-TPP (*n* = 200)	SIL-TEP (*n* = 56)	*p* value
Age (years)	61.10 ± 14.66	59.23 ± 15.02	0.403
Sex			0.837
Male	174	49	
Female	26	7	
BMI (kg/m2)	22.86 ± 2.78	21.97 ± 6.07	0.292
Median ASA	1.77 ± 0.54	1.63 ± 0.52	0.085
Site of hernias			0.02
Left	60	17	
Right	97	35	
Both	43	4	
Main type of hernias			0.420
Indirect	132	41	
Direct	17	7	
Femoral and obturator	6	1	
Combined hernia	3	3	
Previous lower abdominal surgery	36	5	0.102
Open tension-free inguinal hernioplasty	10	3	
Appendicectomy	10	0	
Metrectomy	1	0	
Prostatectomy	1	0	
Laparoscopic colorectum resection	3	0	
Colorectum resection	3	1	
Cesarean	2	0	
Distal gastrectomy	3	0	
Another side of SIL-TPP or SIL-TEP	3	1	

The data are given as the mean ± SD or number, unless otherwise Specified

**Table 2:** The perioperative data for the SIL-TPP and SIL-TEP repair groups

Variable	SILS-TPP (*n* = 200)	SILS-TEP (*n* = 56)	*p* value
Operation time			
Unilateral (min)	81.38 ± 25.32	95.96 ± 28.54	0.001
Bilateral (min)	114.10 ± 34.25	122.75 ± 34.74	0.631
Blood loss	Minimal	Minimal	-
Conversion	0	0	-
Intraoperative complication			0.399
Major bleeding	0	0	
Bowel injury	0	0	
Ductus deferens injury	0	0	
Bladder injury	0	0	
Internal spermatic vessel injury	0		
Minor bleeding	0	0	
Transection of vas deferens	0	0	
Peritoneum or sac tearing	62	13	

**Table 3:** Comparison of postoperative outcomes between SIL-TPP and SIL-TEP.

Variable	SILS-TPP (*n* = 200)	SILS-TEP (*n* = 56)	*P* value
Postoperative hospital stay, days	2.45 ± 1.59	3.20 ± 2.46	0.007
Complications			0.209
Seroma	1	0	
Hematoma	0	0	
Wound infection	0	1	
Incisions liquid exudation	1	2	
Incision hematoma	0	1	
Mesh infection	0	0	
Upper respiratory infection	1	0	
Urinary retention	0	0	
Urinary tract infection	1	0	
Hydrocele of testes	1	0	
Visual analogue scale score (24 h)	2.03 ± 0.76	1.87 ± 0.79	0.180
Umbilical hernia	0	0	-
Recurrence	1	0	-

### Unilateral hernia patient analysis

**Fig. 1:**
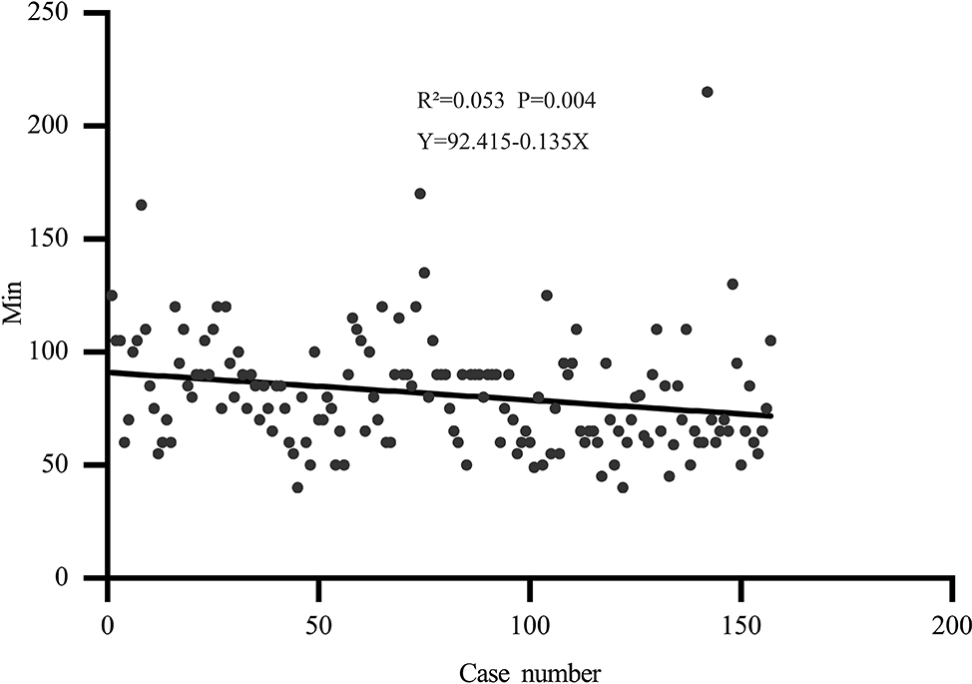
The operation time trend of unilateral side case in SIL-TPP group

**Fig. 2:**
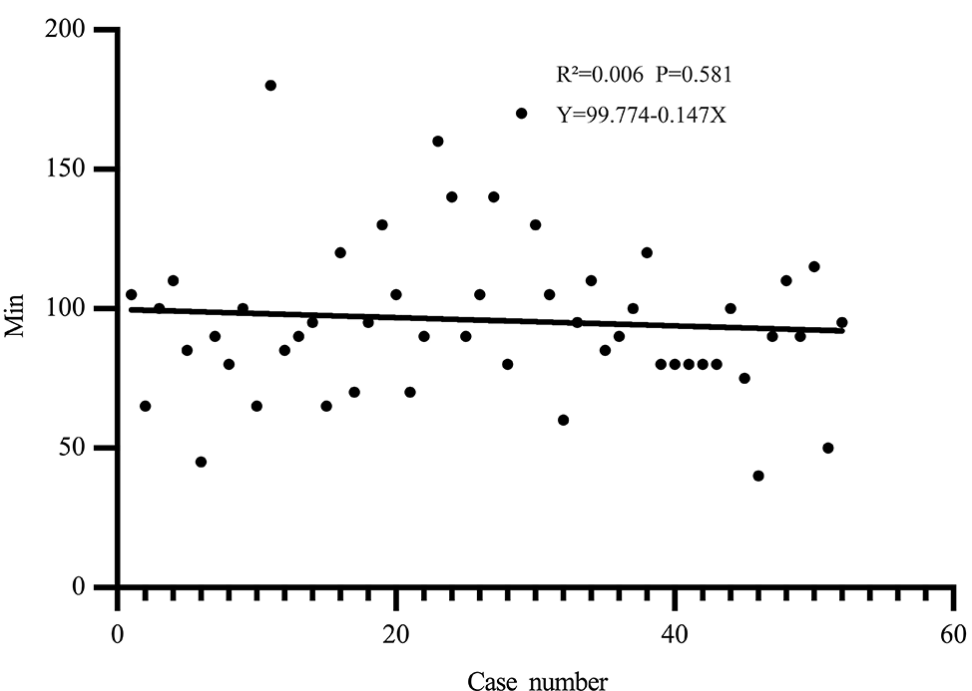
The operation time trend of initial 52 unilateral side case in SIL-TEP group

Unilateral hernia patient characteristics were analysis. Comparative demographics of unilateral hernia patients between SIL-TPP group and SIL-TEP group are described in Table 4. 34 patients and 7 cases received lower abdominal surgery before or suffered incarcerated hernia were regard as time consuming cases respectively. Parameters regarding to age, BMI, median ASA, main type of hernias, patients of left site of hernias and right site of hernias, previous lower abdominal surgery cases and time-consuming cases were comparable (*P* > 0.05). The rate of time-consuming case in SIL-TPP (34/157,21.66%) was high than in SIL-TEP (7/52, 13.46%) group. Comparative perioperative data was shown in Table 5. The mean indirect hernia operative time was significant shorter in the SIL-TPP group than SIL-TEP group (indirect: 81.38 ± 25.33 vs. 95.87 ± 28.54, P: 0.001). The mean direct hernia operative time in the SIL-TPP group was slightly longer than SIL-TEP group without statistical significance (direct: 75.00 ± 24.30 vs. 71.43 ± 15.74, *P* = 0.699). The intraoperative complication rate (*P* = 0.772) and the postoperative complication rate was comparable (no shown). The unilateral hernia operation time trend of two group were shown in Figs. 1 and 2. The results revealed the operation time of two group was decreasing when the treating number increasing. Moreover, the operation time trend in SIL-TPP was liner (Fig. 1: R²=0.053, *P* = 0.004). However, the operation time trend in SIL-TEP was not liner (Fig. 2: R²=0.006, *P* = 0.581).

**Table 4:** The characteristics of the unilateral side hernias patients

Characteristics	SIL-TPP (*n* = 157)	SIL-TEP (*n* = 52)	*p* value
Age (years)	61.68 ± 14.11	58.46 ± 15.26	0.164
Sex			0.921
Male	135	45	
Female	22	7	
BMI (kg/m2)	22.91 ± 2.92	23.22 ± 3.03	0.513
Median ASA	1.77 ± 0.56	1.61 ± 0.53	0.082
Left site of hernias			0.960
Indirect	47	14	
None indirect	13	4	
Right site of hernias			0.166
indirect	84	26	
None indirect	13	8	
Main type of hernias			
Indirect	131	41	0.139
None Indirect	26	11	0.452
Previous lower abdominal surgery	26	5	0.188
Open tension-free inguinal hernioplasty	6	3	
Appendicectomy	7	0	
Metrectomy	1	0	
Prostatectomy	1	0	
Laparoscopic radical resection of colorectal cancer	3	0	
Radical resection of colorectal cancer	2	1	
Cesarean	1	0	
Radical distal gastrectomy of gastric cancer	2	0	
Another side of SIL-TPP or SIL-TEP	3	1	
Time-consuming case	34	7	0.168
Previous lower abdominal surgery^*^	26	5	
Incarcerated hernia	8	2	

* There were 1 case combined with incarcerated hernia

**Table 5:** The perioperative data of unilateral side case between the SIL-TPP and SIL-TEP.

Variable	SILS-TPP (*n* = 157)	SILS-TEP (*n* = 52)	*P* value
Operation time			
Indirect	81.38 ± 25.33	95.87 ± 28.54	0.001
Direct	75.00 ± 24.30	71.43 ± 15.74	0.699
Femoral or obturator	90.83 ± 25.62	-	-
Combined hernia	80.00 ± 13.23	93.33 ± 25.17	-
Blood loss	Minimal	Minimal	
Conversion	0	0	
Intraoperative complication			0.772
Major bleeding	0	0	
Bowel injury	0	0	
Ductus deferens injury	0	0	
Bladder injury	0	0	
Internal spermatic vessel injury	0		
Minor bleeding	0	0	
Transection of vas deferens	0	0	
Peritoneum or sac tearing	45	16	

There were few patients and no comparison

### Initial equal quantity unilateral hernia patient analysis

**Fig. 3:**
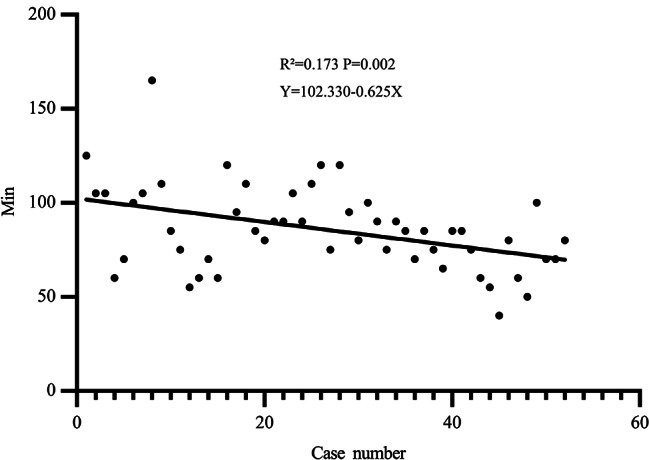
The operation time trend of initial 52 unilateral side case in SIL-TPP group

The parameters of initial 52 unilateral cases of two group were also analysed for comparing thus reflecting actual operation parameters of the two group. The Comparative demographics of two group are described in Table 6. Parameters regarding to age, BMI, median ASA, main type of hernias, patients of left site of hernias and right site of hernias were comparable (*P* > 0.05). The rate of time-consuming cases in SIL-TPP (rate: 19.23% (7/52)) were higher than in SIL-TEP (rate: 7.69% (5/52)) (no shown). The mean operative time of the SIL-TPP group was significant shorter than SIL-TEP group (85.77 ± 22.76 vs. 95.87 ± 28.54, P: 0.049). The rate of peritoneum tearing of SIL-TPP group was significant high than SIL-TEP (*P* = 0.005). The operation time trend of SIL-TPP group was decreasing faster than SIL-TEP group when the treating number increasing (Figs. 2 and 3). The initial 52 unilateral hernia operation time trend in SIL-TPP was also liner (Fig. 3).

**Table 6:** The perioperative comparison data of first 52 unilateral side case between SIL-TPP group and SIL-TEP group

Variable	SILS-TPP (*n* = 52)	SILS-TEP (*n* = 52)	*P* value
Operation time			
Unilateral (min)	85.77 ± 22.76	95.87 ± 28.54	0.049
Blood loss	Minimal	Minimal	
Conversion	0	0	
Intraoperative complication			0.005
Major bleeding	0	0	
Bowel injury	0	0	
Ductus deferens injury	0	0	
Bladder injury	0	0	
Internal spermatic vessel injury	0		
Minor bleeding	0	0	
Transection of vas deferens	0	0	
Peritoneum or sac tearing	28	14	

## Discussion

Inguinal hernia repair is one of the most common surgical procedures. At present, it is still an issue about first-choice surgery for inguinal hernia. Single-incision laparoscopy (SIL) has attracted interest in the past decade, mainly because it improves cosmetic outcomes and reduces trauma [7]. Besides, the safety and efficacy of SIL–TEP for inguinal hernia repair had been verified in a serious of studies [3–5, 8, 9]. It is regretful that almost all SIL-TEP procedure reported before were operated through the space between the front of the posterior sheath and rectus abdominis and then enter the PPS for repairing inguinal hernia. Consideration the characteristics of PPS, we introduced the TPP concept and succeed to repair the inguinal hernia totally through preperitoneal space. Remarkable, our results showed the SIL-TPP is a safe and feasible procedure with acceptable short-term outcomes for inguinal hernia repairation [6]. In current study, the perioperative, short-term, and mid-term outcomes of SIL-TEP and SIL-TPP were compared. The results showed the SIL- TPP has its own advantages when compared to SIL-TEP.

Increasing evidence have verified the safety and feasibility of SIL-TEP, even in elderly patients and patients accepting antithrombotic treatment or suffering incarcerated inguinal hernia [10–12]. In addition, evidence also suggested the intraoperative outcome and postoperative outcome were comparable in SIL-TEP when compared to conventional TEP [9, 13, 14]. In current study, the intraoperative complication rate of SIL-TPP group and SIL-TEP group was 26.0% and 23.21% respectively. Most complications in our study were peritoneum or sac tearing and minor bleeding which did not conduce postoperative morbidity. As we have report before, peritoneum or sac tearing didn’t cause obvious difficulty to operation for its favorable factors in SIL-TPP [6] (Fig. 7). In addition, the postoperative complication rate was low and most of them were minor. Besides, there was no case need to convert to other procedures and reoperate. Consequence, it is believed the safety and short-term outcome of SIL-TPP and SIL-TEP in our study was comparable when compared to previous SIL-TEP and conventional TEP study [5, 8, 9, 14, 15].

**Fig. 4:**
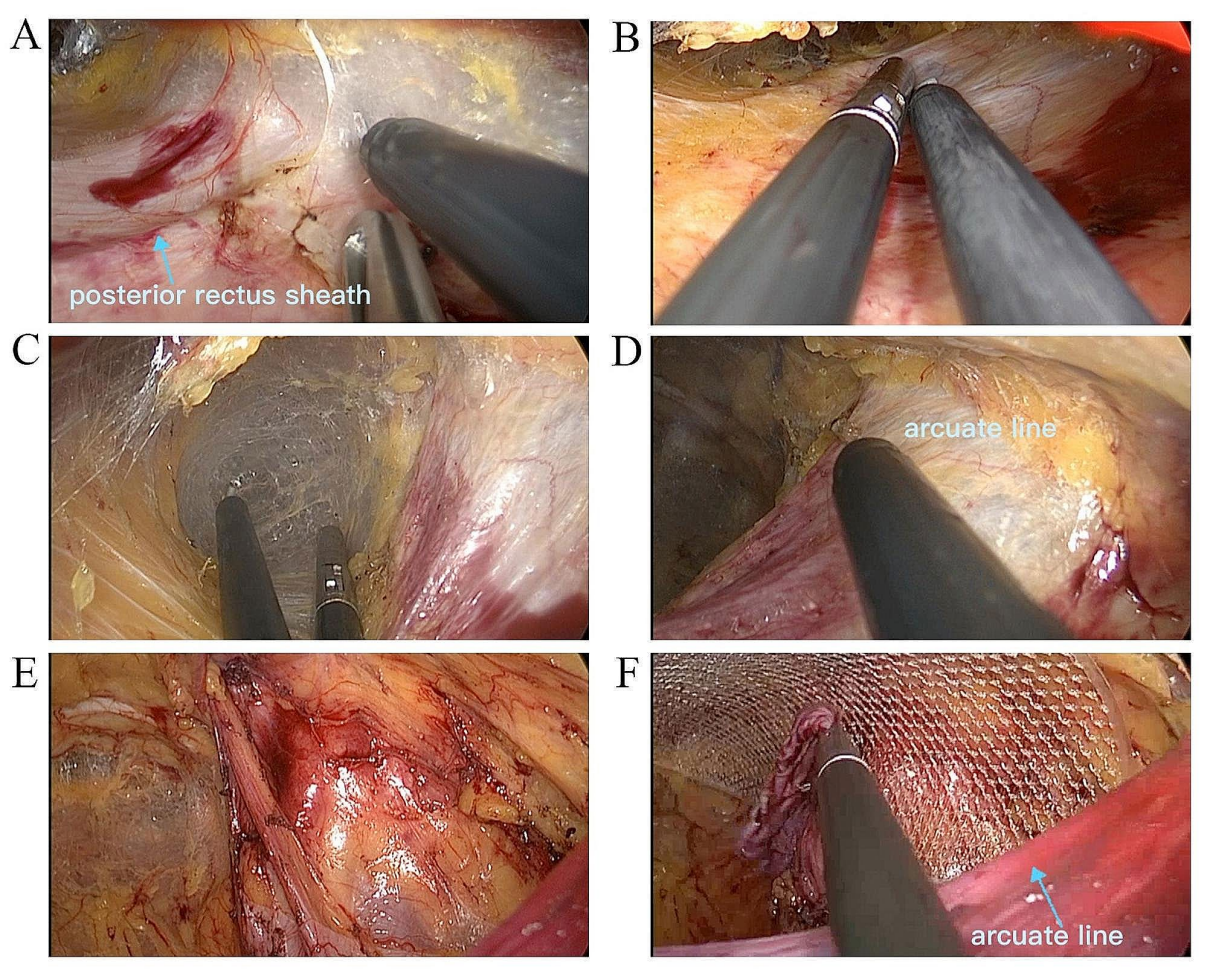
Without peritoneum tearing in SIL-TEP operation. **(A)** Enter the space above the posterior rectus sheath. **(B)** The confined operation space. **(C)** Entering Retzius space. **(D)** The arcuate line makes it difficult to expose the Bogras space. **(E)** The space finished separation **(F)** Arcuate line usually hinders the vision of distant PPS organization structure

**Fig. 5:**
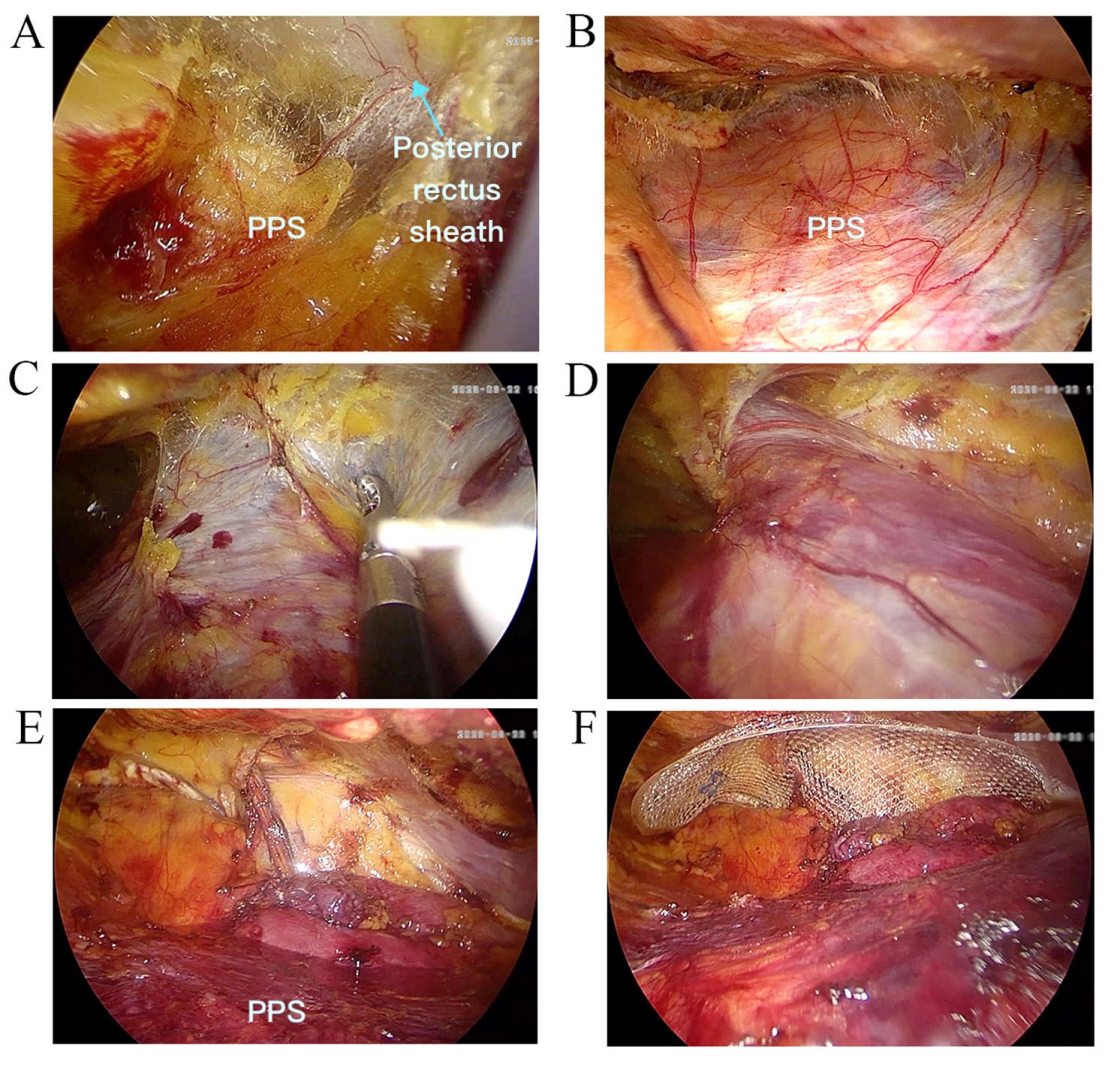
Without peritoneum tearing in SIL-TPP operation. **(A)** Enter the PPS. **(B)** The large operation space. **(C)** easy to entering Bogras space. **(D)** Easy to separat the Bogras space. **(E)** The space finished separation **(F)** The wide visual field and flat peritoneum

**Fig. 6:**
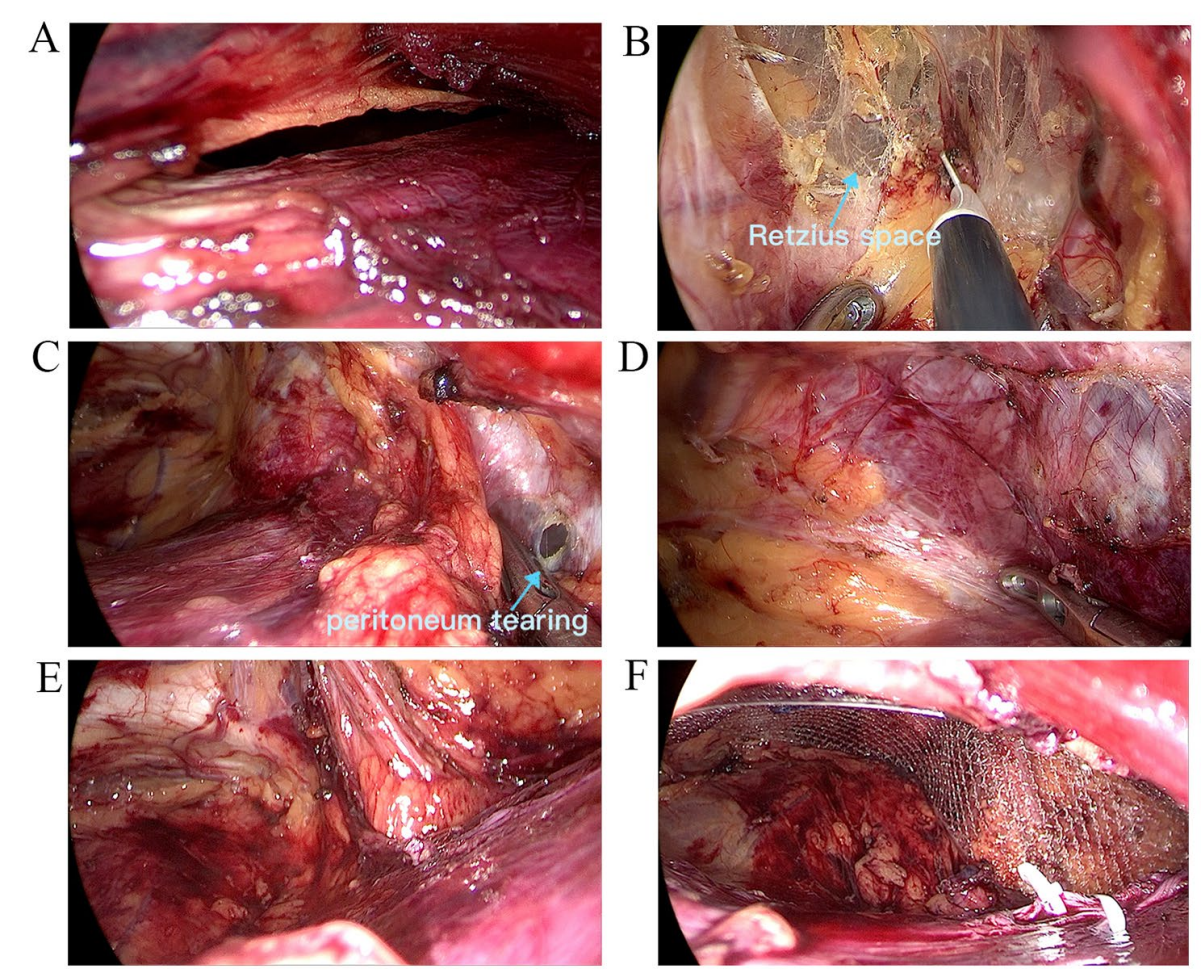
Peritoneum tearing in SIL-TEP operation. **(A)** the operation channel close to incision was scaled down after peritoneum tearing. **(B)** Entering Retzius space simple and smooth. **C** and **D**. Difficult to expose and enter the Bogras space. **E**. The space finished separation. **F**. Mesh placement

**Fig. 7:**
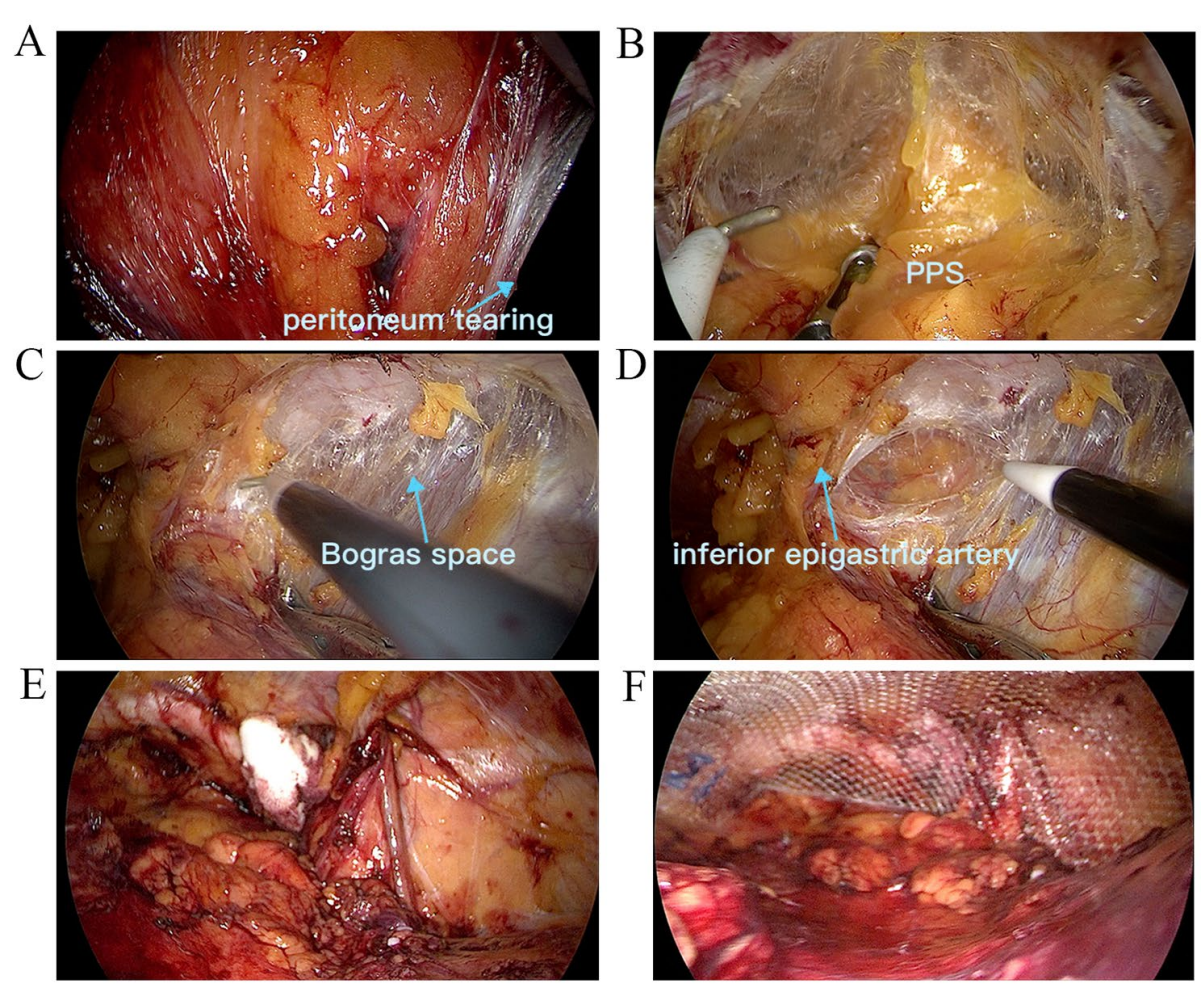
Peritoneum tearing in SIL-TPP operation. **(A)** Peritoneum tearing below the incision. **(B)** Entering Retzius space simple and smooth. **C** and **D**. Easy to expose and enter the Bogras space. **E**. The space after separation. **F**. Mesh placement

Although SIL-TEP owe its difficulties, such as confined operating space, in-line positioning of laparoscope, instruments confliction and so on [16], evidence suggested the mean operative time for unilateral hernia in SIL-TEP was comparable to previous CL TEP studies for experienced surgeon who have overcame the learning period [14, 17]. In addition, selection bias for surgical candidates is also a significant factor affecting the operating time [3, 12, 18]. Though our initial 52 cases mean operating time of SIL-TEP in study is longer than some previous study only operated for primary male inguinal hernia patients or operated by experienced surgeon [5, 13, 14]. After we exclude the discrepancy variable, the operating time of SIL-TEP is comparable when compare to their results [9, 15, 19]. In current study, during SIL-TPP period and SIL-TEP period, almost all inguinal hernia inpatient were recruit into study. Consequently, our results could reduce the influence of selection bias thus typically reflecting the actual operation information in clinical practice. In current study, patients such as previous lower abdominal surgery and incarcerated hernia were regarded as time-consuming cases for these cases will consuming more time on operation. Remarkably, the rate of time-consuming cases in SIL-TPP group was higher than in SIL-TEP group. Interestingly, the mean operating time of SIL-TPP was significant shorter than the mean operating time of SIL-TEP. In order to eliminate the bias of operation number between SIL-TPP and SIL-TEP. The operation time of initial 52 cases of unilateral inguinal hernia between SIL-TPP and SIL-TEP was also compared. The results also suggested the operating time in SIL-TPP group was significant shorter than SIL-TEP group. As our previous description and Tsai et al. opinion [20], establishing single incision in SIL-TEP is a time-consuming procedure. However, establishing the single incision in SIL-TPP needs more steps than SIL-TEP. Hence, the process of setting single incision in SIL-TPP will spend more time than in SIL-TEP. Consequently, it can be concluded that the pure operation time after excluding the time of setting single incision of SIL-TPP would be more significant shorter than SIL-TEP. In addition, our results revealed the operation time trend of SIL-TPP group was decreasing faster than SIL-TEP group when the treating number increasing. In addition, for the operation time in SIL-TPP decreased in linear correlation, we believe the learn curve of SIL-TPP may shorter than SIL-TEP. These distinct phenomenon may partial result from the SIL-TPP procedure advantages and the SIL-TEP procedure disadvantages those we have descript in our previous study before [6]. Remarkably, our results suggest SIL-TPP has distinct advantage in conducting indirect hernia relative to direct hernia when compared to SIL-TEP. Consequence, we believe SIL-TPP maybe a less time-consuming procedure when compared to SIL-TEP, especially for indirect hernia.

Obviously, the operation space is a vital factor for SIL-TPP and SIL-TEP. Owing to the stress of posterior sheath of rectus abdominis, the operation space is confined in SIL-TEP (Fig. 4). Moreover, the peritoneum tearing may further confine the SIL-TEP operation space (Fig. 6). The confined space would make it more difficult in separating Bogros space (Fig. 6). However, owing to the soft peritoneum plus the stress of posterior sheath of rectus abdominis, the SIL-TPP operation space is larger than SIL-TEP in procedure (Fig. 5). Besides, the operation visibility is another vital factor affecting the operation feasibility and safety in SIL-TPP and SIL-TEP. However, arcuate line usually hinders the vision of distant PPS organization structure (Figs. 4 and 6). Besides, in TEP procedure, the variations of the arcuate line often make the preperitoneal working space unfamiliar for surgeon, thus increasing the risks of complications and recurrence [21]. However, in TPP procedure, all procedures are conducted in PPS. Hence, the SIL-TPP procedure was simpler than SIL-TEP procedure for SIL-TPP procedure was handled in the single PPS [6]. Hence, the advantages above were the reason we conduced more SIL-TPP than SIL-TEP in our study. In a short, SIL-TPP is at least as effective as SIL-TEP and we recommend it rather than SIL-TEP for treating inguinal hernia.

In summary, this study has the following limitations. First, it was a retrospective study. Second, the short-term outcome such as peritoneum tearing and seroma were undercounted for the small peritoneum tearing was ignored and lost to follow-up. Third, the number of patients recruited in the study was small and was not equal quantity.

## Conclusions

SIL-TPP hernia repair is also a safe and superior procedure when compared to SIL-TEP. SIL-TPP possess its own distinguished advantages, such as the large and simple space. Moreover, we prefer it rather than SIL-TEP to repair inguinal hernia, especially for indirect hernia. However, large-scale randomized controlled trials comparing SIL-TPP and SIL-TEP are needed to confirm these results.

## Data Availability

The datasets used and/or analysed during the current study are available from the corresponding author on reasonable request.

## Abbreviations

TPP: Totally preperitoneal hernioplasty
TEP: totally extraperitoneal
SIL-TPP: Single incision laparoscopic totally preperitoneal hernioplasty
SIL-TEP: Single incision laparoscopic totally extraperitoneal hernioplasty
TAPP: transperitoneal hernioplasty
PPS: preperitoneal space
CT: Computed Tomography
BMI: Body Mass Index
ASA: American Society of Anesthesiologists
